# Knockout of aminopeptidase N3 confers resistance to low concentrations of *Bacillus thuringiensis* Cry4Ba protoxin in *Aedes aegypti*

**DOI:** 10.1186/s13071-026-07360-x

**Published:** 2026-03-21

**Authors:** Xiaozhen Yang, Wanting Huang, Jiajia Wei, Xiaoxuan Xu, Jackson Champer, Lingling Zhang, Junxiang Wang

**Affiliations:** 1 Fuzhou Institute of Oceanography, School of Materials and Chemistry Engineering, Minjiang University, Fuzhou, 350108 China; 2 College of Life Science, Fujian Agriculture and Forestry University (FAFU), Fuzhou, 350002 China; 3 Center for Bioinformatics, Center for Life Sciences, School of Life Sciences, Peking University, Beijing, 100871 China

**Keywords:** *Aedes aegypti*, Aminopeptidase N, *Bacillus thuringiensis*, Cry toxin, CRISPR/Cas9

## Abstract

**Background:**

*Bacillus thuringiensis* is widely employed for biological control. It can effectively suppress populations of various mosquito species, including *Aedes aegypti*. However, the precise mechanism underlying the action of Cry protein produced by *Bacillus thuringiensis* on *Ae. aegypti* remains elusive. On the basis of our previous research findings, five *Aedes* aminopeptidase N proteins (AeAPNs) were identified from the brush border membrane vesicles (BBMV) of *Ae. aegypti* that could bind to Cry4Ba or Cry11Aa. Further analysis confirmed that AeAPN1 and AeAPN2 are not functional receptors for these proteins. In this study, we investigated an additional aminopeptidase N (*AeAPN3*, AAEL012774) as a potential binding receptor for Cry proteins.

**Methods:**

Comprehensive bioinformatics analysis involving whole-genome screening, genetic mapping, structural characterization, phylogenetic analysis, and spatiotemporal expression profiling were used to identify *Ae. aegypti* aminopeptidase N homologs. Ligand blotting and enzyme-linked immunosorbent assay (ELISA) were used to measure binding affinity to Cry4Ba. To elucidate its functional role as a potential receptor mediating Cry4Ba activity in *Ae. aegypti* midgut cells, *AeAPN3* was knocked out with CRISPR/Cas9 technology.

**Results:**

A total of 29 homologs of *Ae. aegypti* aminopeptidase N were identified in this study. Then, we expressed GST–APN3 fusion protein in *E. coli* and found that it had high-affinity binding to Cry4Ba protein (*K*_*d*_ = 20.53 nM). Mosquito larvae had approximately threefold higher resistance against Cry4Ba after* AeAPN3* knockout, indicating its significant involvement as an active receptor mediating Cry4Ba activity.

**Conclusions:**

Overall, this study provides a foundation for elucidating the specific larvicidal mechanisms of *Bacillus thuringiensis* (Bt) against mosquito populations.

**Graphical Abstract:**

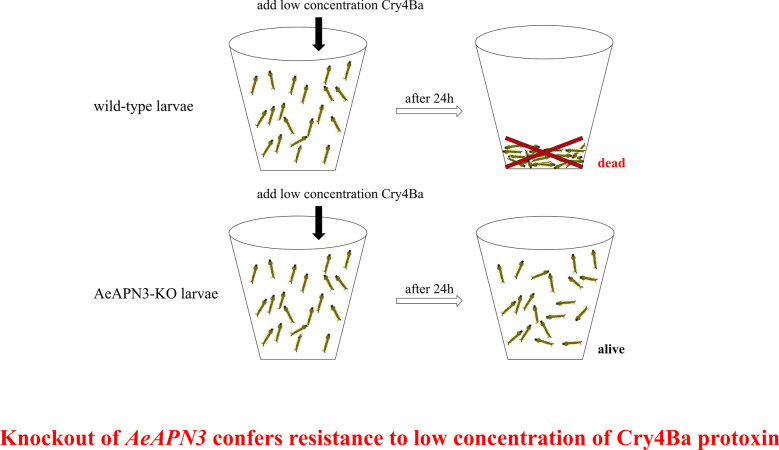

**Supplementary Information:**

The online version contains supplementary material available at 10.1186/s13071-026-07360-x.

## Background

*Aedes aegypti* is the principal arthropod vector responsible for the transmission of several medically significant arboviral infections, most notably dengue fever [1, 2]. Recent epidemiological data from the World Health Organization (WHO) reveal that approximately 4.1 billion people across 132 endemic countries are at substantial risk of dengue virus exposure [3]. The absence of universally approved prophylactic vaccines underscores the critical necessity for implementing effective vector control to disrupt disease transmission. Under the guidance of the WHO, strategies such as “integrated and sustainable vector management” have yielded improvements in mosquito control [3, 4]. Presently, biological control has gained international recognition as a preferred measure for disease and pest prevention and control. *Bacillus thuringiensis* (Bt) has emerged as the most extensively studied and widely applied biocontrol microorganism owing to its high insecticidal activity, broad-spectrum efficacy, strong target specificity, and nontoxicity toward humans and animals [5–7].

Bt synthesizes a diversity of proteins exhibiting insecticidal properties throughout its vegetative, growth, and sporulation phases. The primary insecticidal protein is known as crystal protein (Cry) toxin, while certain strains also produce cytolytic proteins (Cyt toxin) and vegetative insecticidal protein (VIP) [7, 8]. Upon ingestion by the target insect, the Cry prototoxin undergoes dissolution in the alkaline environment of the intestine and subsequent activation through protease hydrolysis [8]. This process involves the removal of nontoxic regions from both N- and C-termini to generate Cry-active monomers. These monomers traverse the peritrophic membrane surrounding the intestinal lumen and interact with receptor proteins on brush border membrane vesicles of epithelial cells [8]. Toxin-receptor binding results in extensive epithelial cell death, giving rise to intestinal ulceration or sepsis and culminating in insect mortality. On the basis of these findings from in vitro and in vivo experiments conducted on different insects, it is generally believed that aminopeptidase N (APN), alkaline phosphatase (ALP), cadherin-like (CAD), and ATP-binding cassette transporter subfamily C are among the main receptor proteins involved [7].

APN is a widely distributed ectopeptidase protein belonging to the M1 zinc metalloprotease family, found in animals, plants, and microorganisms. In insects, Glycosylphosphatidylinositol (GPI)-anchored APN membrane proteins serve as crucial binding receptors for Cry toxins [9]. In vitro binding experiments have demonstrated that APN proteins from various insects can bind to different Cry toxins, including *Manduca sexta *[10], *Trichoplusia ni *[11], *Anopheles quadrimaculatus *[12], *Anopheles gambiae *[13], *Ae. Aegypti *[14–16], and other Lepidoptera [17–20] and Coleoptera [21] insects. However, recent studies utilizing CRISPR/Cas9-mediated knockout of certain APN genes have revealed no alteration in sensitivity toward corresponding Cry toxins [22–24]. Therefore, further validation is required to elucidate the significance of APN in mediating the mechanism of action of Bt.

*Bacillus thuringiensis* subsp. *israelensis* (Bti) secretes a range of proteins that specifically target mosquito larvae, including Cry4Aa, Cry4Ba, Cry10Aa, Cry11Aa, Cyt1Aa, Cyt1Ca, and Cyt2Ba [25]. Among these, Cry4Ba and Cry11Aa have been found to exhibit activity against *Ae. aegypti* larvae [25]. However, the precise role of APN in the mechanism of action of these two proteins on *Ae. aegypti* larvae remains unclear. In our previous study utilizing GST-pull down and co-immunoprecipitation (Co-IP) techniques from *Ae. aegypti* brush border membrane vesicles, we identified five APN proteins (APN1–APN5) that interact with either Cry4Ba or Cry11Aa [14]. Knocking out APN1 and APN2 did not impact the insecticidal activity of Cry4Ba and Cry11Aa [22].

To investigate whether other APN genes are involved in this process, we initially characterized the genomic structure features of APNs in *Ae. aegypti* through whole-genome identification as well as transcriptomic analysis of APN transcripts at different developmental stages and in various tissues, ultimately focusing on APN3. In our previous identification of Bti proteins binding proteins within the midgut epithelium of *Ae. aegypti* mosquitoes, we found that AeAPN3 exhibits specific binding affinity to the Cry11Aa protein [14]. We then investigated an interaction between an AeAPN3 protein and Cry4Ba using brush border membrane vesicles from *Ae. aegypti*. The binding affinity between the Glutathione S-transferase (GST)–APN3 fusion protein expressed in prokaryotic cells and Cry4Ba was determined through ligand blotting, and an enzyme-linked immunosorbent assay (ELISA) was performed to determine their binding affinity. Finally, CRISPR/Cas9 was employed to construct an *AeAPN3*-KO gene knockout strain, confirming that AeAPN3 functions as the functional receptor mediating the low-dose activity of Cry4Ba.

## Methods

### Mosquito strains and rearing

The wild-type strain of *Ae. aegypti* (Haikou strain) was provided by the Fujian International Travel Health Care Center (Fuzhou, Fujian, China). The EXU strain is a Cas9 knock-in strain derived from the wild-type strain through microinjection [26]. Strains were reared for over 70 generations and 10 generations, respectively, without exposure to Bt protoxins. All *Ae. aegypti* strains were maintained under controlled conditions at 26 ± 1 ℃, with a relative humidity of 83% ± 3% and a photoperiod of 14:10 h (light:dark). *Ae. aegypti* larvae were fed goldfish feed (Tetra, Germany), while adult mosquitoes received a diet consisting of a 10% sucrose solution. Female mosquitoes were provided with sterile defibrinated bovine blood for reproduction.

### Bt strain and purification of Cry4Ba and Cry11Aa proteins

The Bt strains producing recombinant Cry4Ba protein (pCG6-Cry4Ba) and Cry11Aa protein (pCG6-Cry11Aa) were provided by Dr. Sarjeet R. Gill’s laboratory, University of California, Riverside. Bt bacteria were cultured in 1/2 Luria–Bertani (LB) medium supplemented with erythromycin at a final concentration of 12.5 μg/mL, and incubated at 30 °C. Once the crystal inclusions were fully released, the culture medium was removed, and the pellets were washed three times with 1 M NaCl plus 0.03% Triton X-100, followed by distilled water rinses. The Cry4Ba was purified by the isoelectric point precipitation method as described previously [27], and protoxin was solubilized in alkaline buffer (50 mM Na_2_CO_3_/NaHCO_3_, pH 10.5). In our preliminary experiments, we observed that Cry11Aa protoxin purified via the standard isoelectric precipitation method lost its larvicidal activity, likely due to denaturation at high pH. Therefore, for Cry11Aa purification, density gradient centrifugation using sucrose solution was performed according to previous protocols [28], followed by solubilization of the protoxin in distilled water.

### Sequence analysis of *AeAPN*

The genomic data of *Ae. aegypti* (AaegL5.2), *Culex quinquefasciatus* (CpipJ2.4) and *An. gambiae* (AgamP4.12) were downloaded from the VectorBase database (https://www.vectorbase.org). The R language package biomaRt was utilized to extract the DNA sequences, complementary DNA (cDNA) sequences, and amino acid sequences of the APN gene family from three genomes. Additionally, relevant gene annotation information was extracted from both VectorBase and National Center for Biotechnology Information (NCBI) databases (https://www.ncbi.nlm.nih.gov). To further investigate the function of APN, the genomic structure of APN genes was visualized using MapGene2Chrom version 2.1 (http://mg2c.iask.in/mg2c%5Fv2.1/), while features of the amino acid sequence were analyzed by SMART (http://smart.embl-heidelberg.de). SignalP 5.0 (http://www.cbs.dtu.dk/services/SignalP/) was applied for predicting the N-terminal signal peptide of APN, whereas transmembrane domain analysis of APN was conducted using TMHMM Server version 2.0 (http://www.cbs.dtu.dk/services/TMHMM/). Furthermore, GPI-anchored points of APN were predicted utilizing big-PI Predictor (https://mendel.imp.ac.at/gpi/gpi_server.html), PredGPI predictor (http://gpcr2.biocomp.unibo.it/gpipe/pred.htm), and KohGPI (http://gpi.unibe.ch). Finally, NetNGlyc 1.0 Server (http://www.cbs.dtu.dk/services/NetNGlyc/) and NetOGlyc 4.0 Server (http://www.cbs.dtu.dk/services/NetOGlyc/) were employed for predicting N-glycosylation sites and O-glycosylation sites in APN.

The ClustalW program of MAGAX 10.1.7 was used to perform multiple sequence alignment for the APN gene family of *Ae. aegypti*, while neighbor joining was utilized for evolutionary tree clustering analysis, selecting the default parameter. The conserved domains, motifs, and protein sequence conduction were visualized using TBtools software. Additionally, further phylogenetic analysis of APN gene families in three pathogenic mosquitoes was performed, and the resulting evolutionary tree was modified using iTOL (https://itol.embl.de).

### Spatiotemporal expression patterns of APNs in *Ae. aegypti*

The transcriptome data of *Ae. aegypti* at different developmental ages were obtained from Matthews et al.’s study [29], including 1st to 4th instar larva, pupa, and male and female adults. RNA was extracted separately from the alimentary tracts (ATs) and bodies minus alimentary tracts (BMATs) of 20 4th instar larvae, followed by transcriptome sequencing conducted by Biomarker Technologies company. To define tissue-specific or stage-enriched expression, we applied the following criteria: (1) gut-enriched genes exhibited ≥ fivefold higher transcripts per million (TPM) in ATs than in BMATs, with TPM ≥ 10 in ATs and false discovery rate (FDR) < 0.05; (2) stage-enriched expression required ≥ fivefold higher TPM compared with other developmental stages under the same FDR threshold; and (3) sex-biased expression was defined as ≥ twofold difference between males and females with FDR < 0.05. Differential expression analysis was performed using DESeq2 (version 1.34.0). The temporal and spatial expression patterns of the APN genes in *Ae. aegypti* were classified and mapped using TBtools software.

### Prokaryotic expression of AeAPN3 protein

The synthesized cDNA was used as a template to amplify AeAPN3 complete coding sequence (CDS) of its single isoform with specific primers (Additional File 1: Supplementary Table S1). After confirmation by sequencing, the resulting polymerase chain reaction (PCR) products were then cloned into the pCold-GST vector using the In-Fusion Snap Assembly Master Mix (Takara, Japan) according to the manufacturer’s instructions. Subsequently, the assembly product was transformed into *E. coli* JM109 cells (Takara, Japan). For protein expression, the pColdAeAPN3 recombinant plasmid was further transformed into *E. coli* BL21 (DE3) cells (Takara, Japan). A 1-mL volume of bacterial suspension was inoculated into 100 mL of LB medium (containing 100 μg/mL ampicillin) and incubated at 37 °C with shaking until the optical density (OD)_600_ reached 0.6–0.8. It was then placed on ice for 30 min, and isopropyl-β-d-thiogalactopyranoside (IPTG) (Beyotime, China) was added to a final concentration of 0.8 mM. It was shaken at 15 °C and 200 rpm for 24 h to express the protein. Purification of AeAPN3 protein was achieved through a glutathione sepharose 4B chromatographic column. To confirm the expression of recombinant AeAPN3 protein, western blot analysis was performed using an anti-GST antibody (TransGen, China).

### Binding analysis of AeAPN3 protein to Cry4Ba protein

Ligand blot assays and binding enzyme-linked immunosorbent assays (ELISAs) were conducted to assess the binding affinity between AeAPN3 protein and Cry4Ba protein. For ligand blot assays, equal amounts of GST–APN3 protein, GST tag protein and Cry4Ba crystal protein were separately separated by sodium dodecyl sulfate–polyacrylamide gel electrophoresis (SDS–PAGE) and transferred onto a polyvinylidene difluoride (PVDF) membrane by wet transfer apparatus. After overnight blocking at 4 ℃ in PBST buffer containing 5% bovine serum albumin (BSA), the Cry4Ba protoxin (Cry4Ba:PBST = 1:150) was added to the above blocking buffer at 25 ℃ for 2 h. After incubation, the membrane was washed three times with phosphate-buffered saline with Tween-20 (PBST) for 15 min each time. Then, the membrane was hybridized with the primary antibody anti-Cry4Ba antiserum (added to the blocking buffer at 1:3000 dilution) at 25 ℃ and 50 rpm for 2 h, followed by three washes and subsequent incubation with alkaline phosphatase (AP)-conjugated secondary antibody (1:1000) for 2 h. After another round of washing three times, the membrane was visualized with BCIP/NBT Alkaline Phosphatase Color Development Kit.

For ELISAs, each well of the 96-well ELISA plates was coated overnight with 5 μg AeAPN3 protein in a final volume of 100 μL of PBS at 4 ℃ and blocked by blocking buffer at 37 ℃ for 2 h. The plate was washed three times with 200 μL of PBST. Then, different concentrations, ranging from 0 to 320 nM, of Cry4Ba protoxins were independently added to wells in a final volume of 100 μL of blocking buffer (PBST, 5% BSA). After incubating at 37 ℃ for 1 h, the plate was washed three times with PBST and treated with primary antibody anti-Cry4Ba antiserum (added to the blocking buffer at a dilution ratio of 1:10,000) for 1 h at 37 ℃. Following this step, the plate was washed three times using Tris-buffered saline Tween (TBST) with 0.1% Tween-20 and incubated with a horseradish peroxidase (HRP)-conjugated streptavidin antisera (1:3000) for 1 h. After washing three times with TBST, a volume of 100 μL of TMB was added to each well and incubated in the dark for 10 min at 37 °C. Finally, 50 μL/well of 2 M H_2_SO_4_ was added to terminate the reaction, and the plate was sent for colorimetry to measure the absorbance at 450 nm using a microplate reader. The ELISA binding plots were generated in GraphPad Prism 8.0, and the *K*_*d*_ values were calculated using Curve Expert 1.4.

### Embryo microinjection and screening of *AeAPN3 *knockout mutants

The CRISPR/Cas9 target sites were designed in the fourth exon of *AeAPN3* gene using CRISPOR program (http://crispor.tefor.net/). Potential off-target effects were evaluated by Cas-OFFinder (http://www.rgenome.net/cas-offinder/). The sgRNA was synthesized and purified in vitro using HiScribe™ T7 High Yield RNA Synthesis Kit (America, NEB) with specific primers (Additional File 1: Supplementary Table S1). Additional Cas9 protein (to ensure the efficiency of the knockout) and sgRNA were microinjected into fresh exuCas9-kmo embryos (the phenotype was red fluorescence and white eyes, and the genotype was DsRed^+^ KMO^−^) [26]. Genomic DNA was extracted from a single leg of each individual mosquito to enable subsequent survival and reproduction, facilitating the identification of gene mutants until a homozygous strain (*AeAPN3*-KO carrying DsRed^+^ and KMO^–^) was established.

Then, phenotypic observation, fluorescent screening, and gene sequencing of *AeAPN3*-KO with DsRed^+^ KMO^−^ strain were performed to select individuals with nonfluorescent and normal eyes for establishing a new *AeAPN3* gene knockout homozygous line (*AeAPN3*-KO), excluding any potential influence of endogenous expression of the CRISPR/Cas9 system and deletion of *kmo* on subsequent functional verification. Other details are as described previously [22]. In the subsequent breeding of the line, five *Aedes* mosquitoes were randomly selected for mixed PCR and sequencing to ensure the genetic homozygosity of the knockout line.

### Bioassay of Bti Cry protoxins

Susceptibility to Bti Cry4Ba and Cry11Aa toxins was determined for different strains of *Ae. aegypti* as previously described [30]. The wild-type and *AeAPN3*-KO strain of *Ae. aegypti* were fed to the beginning of the 4th instar, and 25 larvae were transferred to 20 mL of filtered water. Then, Cry4Ba or Cry11Aa crystal proteins were added at concentrations according to previous experiments. Six concentrations were set, with three biological replicates performed. Larval mortality was calculated 24 h later. The bioassay was independently repeated three times to ensure experimental reproducibility.

### Data analysis

The lethal concentration required to kill 50% of larvae (LC_50_) was calculated using PoloPlus software. Statistical analysis of mortality was performed by GraphPad Prism9 with Mann–Whitney *U* test.

## Results

### Chromosome localization of APN members in *Ae. aegypti*

**Fig. 1:**
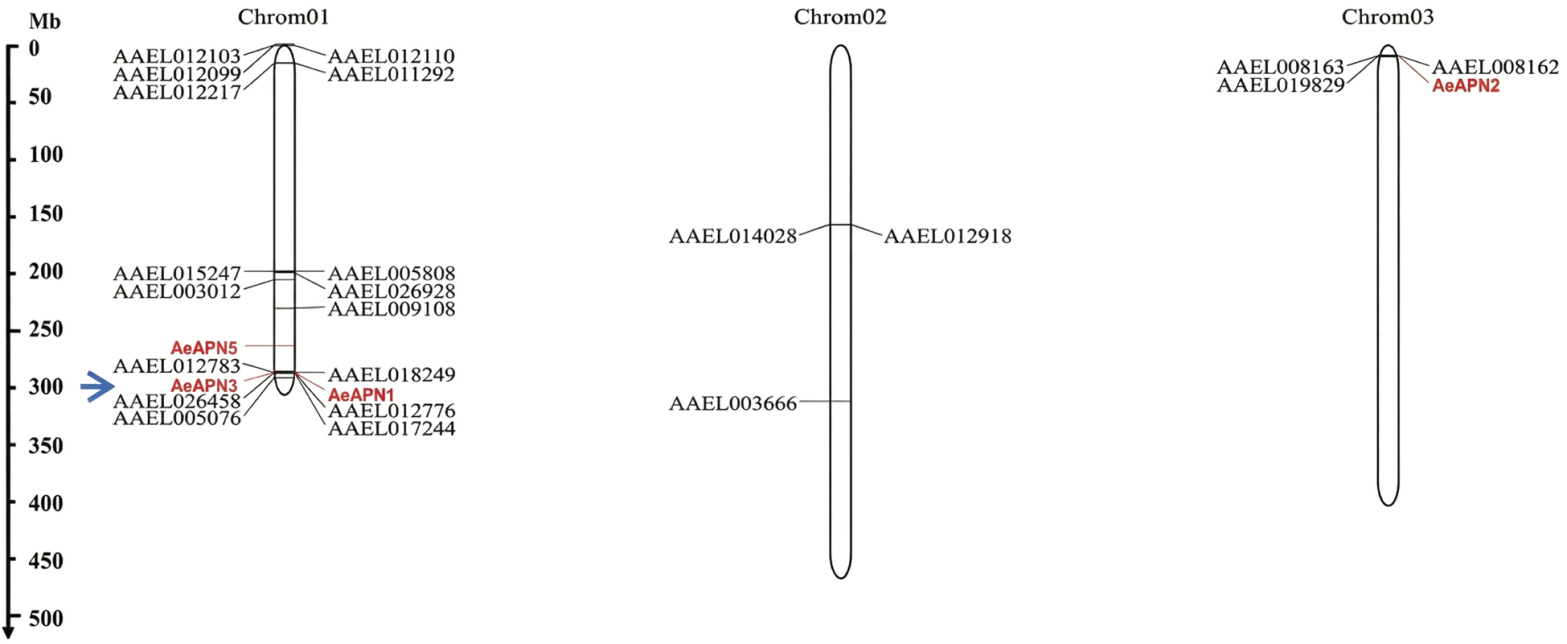
Physical map of APN genes in *Ae. aegypti* chromosomes. Cry-binding APN isoforms are marked in red. The blue arrow indicates the location of *AeAPN3*. *AeAPN4*, AAEL020609, and AAEL022733 reside on unplaced scaffolds and are not displayed

A total of 29 APNs were identified from the *Ae. aegypti* genome on the basis of the conserved APN core motif. Among these, in vitro interaction experiments have demonstrated that *APN1* (AAEL012778), *APN2* (AAEL019828), *APN3* (AAEL012774), *APN4* (AAEL005821), and *APN5* (AAEL019536) were capable of binding to Bti Cry toxins [14]. Chromosomal localization analysis revealed that 26 *AeAPN* genes were distributed on three chromosomes (1, 2, and 3). However, *APN4*, AAEL020609, and AAEL022733 were mapped to unassigned genomic scaffolds (NIGP01002224, NIGP01000461, and NIGP01001046, respectively), and were consequently excluded from the chromosomal distribution analysis. Spatial distribution analysis indicates chromosomal preference, with 73.1% (19/26) of chromosomally anchored APN genes residing on chromosome 1. These genes are predominantly clustered in subtelomeric regions or nearby. The APN genes of *Ae. aegypti* predominantly occur in the form of gene clusters. For instance, *APN1*, *APN3*, and AAEL012776 are arranged consecutively on chromosome 1. Similarly, on chromosome 3, there is a gene cluster consisting of *APN2*, AAEL019829, AAEL008162, and AAEL008163. This clustered genomic organization indicates that the APN gene family of *Ae. aegypti* has undergone multiple evolutionary replication events (Fig. 1).

### Gene structures and conserved motifs analysis of AeAPNs

Bioinformatic characterization of AeAPN protein structures revealed that 11 AeAPNs have predicted GPI anchoring sites at the C-terminal, including four Bti-binding proteins (APN1, APN2, APN3, and APN5). Comparative analysis of N-terminal modifications demonstrated commonalities and variations. While all five AeAPNs (APN1–5) possessed identifiable signal peptides and N-glycosylation sites, only four had O-glycosylation sites. APN1 exhibited a unique absence of O-glycosylation modification. Notably, APN5 was predicted as the most extensively glycosylated isoform, containing 7 N-linked and 13 O-linked glycosylation sites (Additional File 1: Supplementary Table S2). Phylogenetic reconstruction of *Ae. aegypti* APNs revealed substantial sequence divergence across family members, indicative of functional diversification through evolutionary processes (Fig. 7). Nevertheless, conserved essential domains were preserved in all five APN paralogs (APN1–5), including the glutamine-activated zinc-binding motif (GAMEN) and the canonical zinc coordination triad (HEXXHX18E) (Fig. 7 and Additional File 1: Supplementary Fig. S1). This structural conservation suggests maintenance of core enzymatic functions despite sequence heterogeneity.

**Fig. 2:**
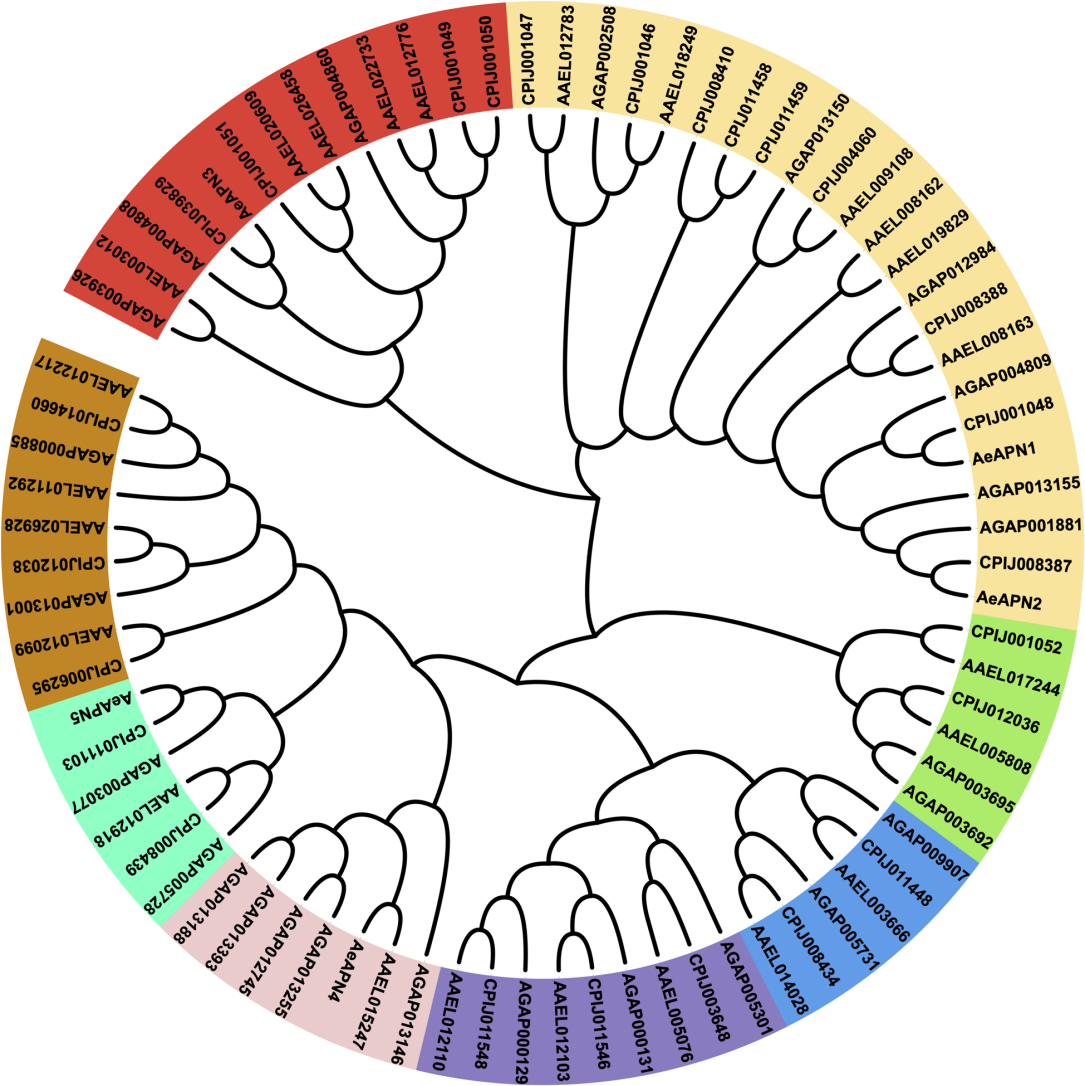
The gene structure and conserved motif analyses of *AeAPN* genes. **A** The phylogenetic relationships of AeAPN proteins. **B** Conserved motif distribution of AeAPN proteins. The different conserved motifs are represented by different colored boxes. **C** Gene structures of *AeAPN* genes. The untranslated region (UTR), CDS, and introns are indicated by red boxes, blue boxes, and black lines, respectively

### Identification and phylogenetic analysis of AeAPNs

**Fig. 3:**
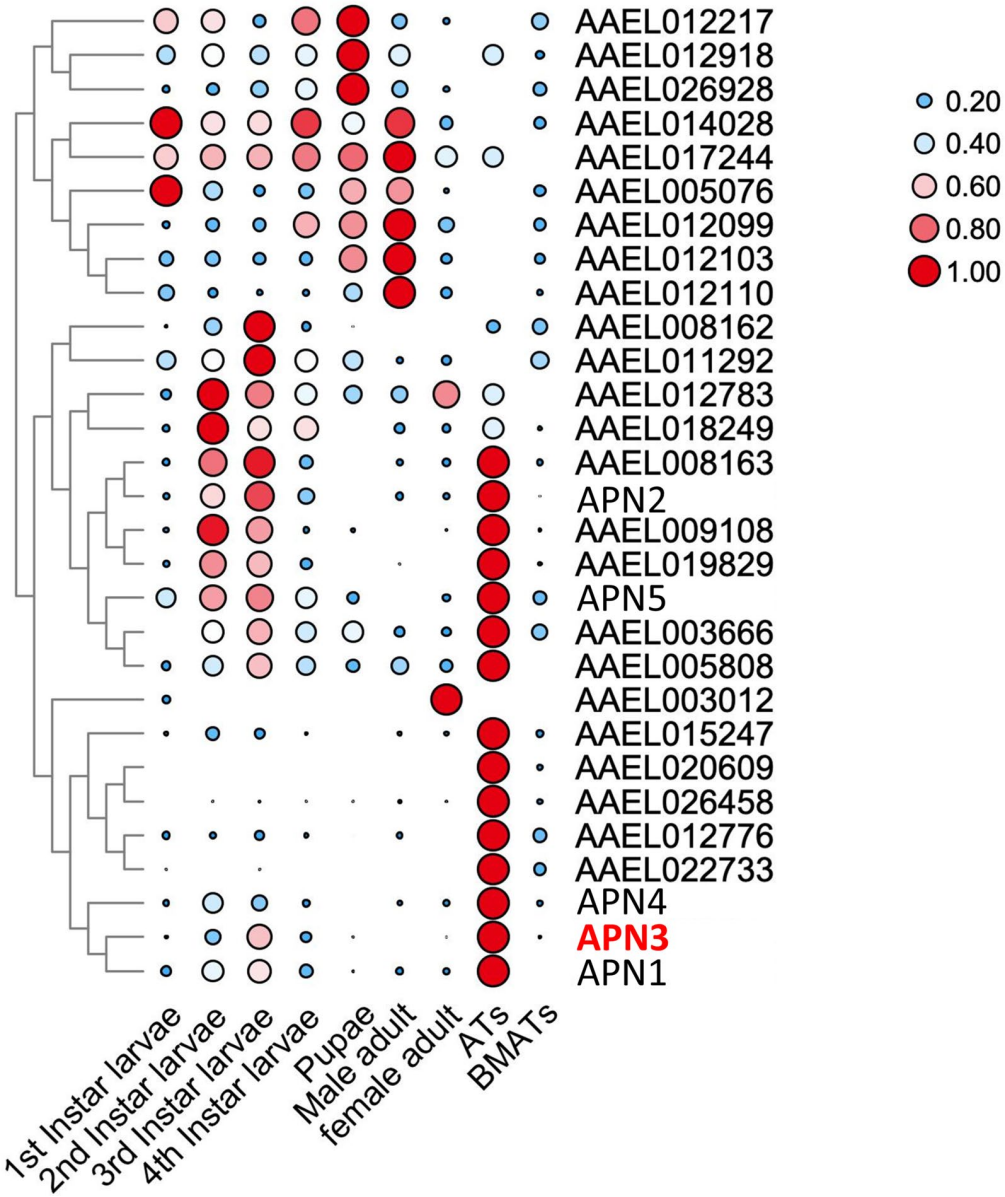
Phylogenetic analysis of APN subunits from three mosquitoes. Phylogenetic tree constructed using amino acid sequences of *Ae. aegypti*, *C. quinquefasciatus*, and *An. gambiae*. The tree was inferred using the neighbor-joining method based on a multiple sequence alignment generated by ClustalW. Bootstrap support values (1000 replicates) are shown at major nodes. The APN proteins are clustered into eight subgroups, marked by different colors, each corresponding to a monophyletic clade with bootstrap support ≥ 90%

To elucidate evolutionary dynamics within the AeAPN gene family, we performed a comparative phylogenetic analysis using full-length protein sequences from three medically important mosquito genera: *Ae. aegypti*, *Culex quinquefasciatus*, and *An. gambiae*. The phylogenetic tree revealed eight well-defined clades (Fig. 3), demonstrating conserved phylogenetic distribution patterns across these dipteran species. Notably, interspecific sequence homology consistently exceeded intraspecific homology levels, suggesting that the diversification events within the APN gene family predate the speciation processes of contemporary mosquito genera. Particularly significant was the phylogenetic segregation of Bti toxin-binding receptors (APN1–5), which occupied distinct clades with substantial branch length divergences (Fig. 3). This topological separation implies both significant divergence in secondary structural configurations and potential functional differentiation among these receptor subtypes.

### Expression pattern analysis of AeAPN gene family

**Fig. 4:**
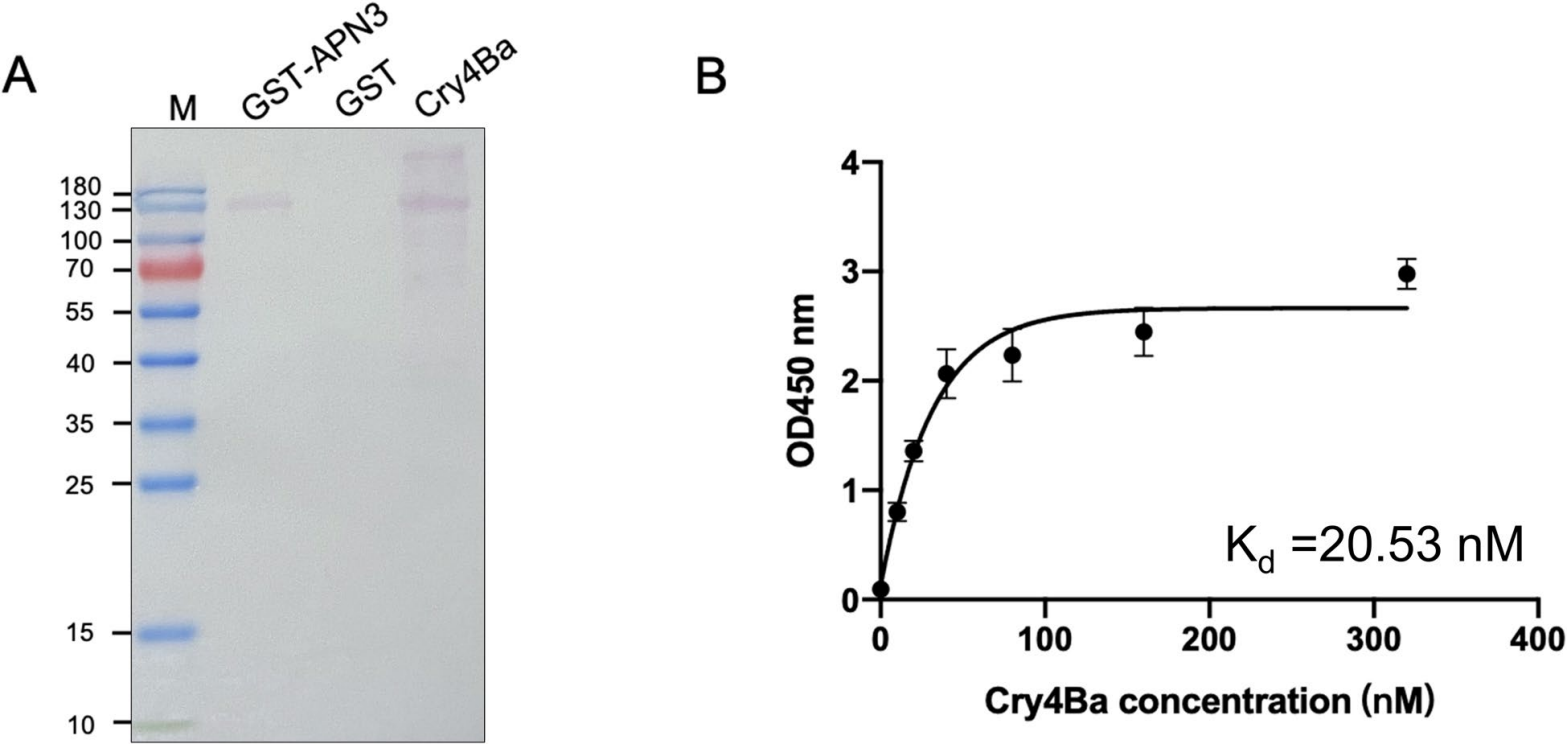
Spatial and temporal expression of APN genes in *Ae. aegypti*. Expression patterns of the *AeAPN* genes in the 1st-instar larvae (L1) to the 4th-instar larvae (L4), pupal stage, male adult, female adult, intestinal tissues (ATs), and extra-intestinal tissues (BMATs). Expression levels are shown on the basis of bubble size and color

Transcriptomic quantification of *AeAPN* gene expression across developmental stages [1st-instar larvae (L1), 2nd-instar larvae (L2), 3rd-instar larvae (L3), 4th-instar larvae (L4), pupal stage, male adult, and female adult] and tissue compartments (intestinal and extra-intestinal tissues) revealed distinct spatial–temporal regulation patterns. Specifically, 15 *AeAPN* genes demonstrated gut-specific expression, while 9 *AeAPNs* exhibited peak transcriptional activity in the L3 stage, including APN1–3 and 5 (Fig. 4). These expression patterns suggest that APNs may contribute to digestive functions in larval midguts. These intestinal-enriched APNs, particularly APN1–3 and 5 that were previously identified as Bti-binding proteins, may serve as candidate receptors involved in Bti toxin susceptibility in *Ae. aegypti*. Furthermore, three APN isoforms showed significant upregulation during pupal metamorphosis (Fig. 4), suggesting a potential role in tissue remodeling. Intriguingly, six male-biased and two female-biased APNs displayed adult stage-specific expression patterns (Fig. 4), implying possible involvement in sex-specific physiological processes such as post-eclosion development, nutrient metabolism, or xenobiotic detoxification.

### Ligand blotting detection and ELISA binding assay of AeAPN3 protein

For the five potential Bti functional receptors (APN1–APN5) identified in the initial study, our previous research confirmed that APN1 and APN2 are not the major functional receptors through knockout experiments [14, 22]. Our gene family analysis indicated that APN4 is located on an unanchored scaffold (NIGP01002224) that has not yet been assigned to a specific chromosome, and the APN5 gene lacks a predicted GPI-anchor attachment site. Given these structural and functional considerations, AeAPN3 emerged as the most suitable candidate for further functional characterization.

Previous co-immunoprecipitation (co-IP) and pull-down assays demonstrated that AeAPN3 interacts with Cry11Aa [14]. To systematically characterize the potential interaction between AeAPN3 and Cry4Ba protein, we cloned the CDS of AeAPN3 into a pColdTMGST prokaryotic expression vector (Additional File 1: Supplementary Fig. S2A) for expressing AeAPN3 protein in *E. coli* BL21(DE3) competent cells. Following nickel-affinity chromatography purification, approximately 0.8 mg of recombinant protein was obtained per liter of culture. The recombinant GST-tagged AeAPN3 protein was subjected to SDS–PAGE analysis, which confirmed the production of a predominant protein band with an apparent molecular mass of ~128 kDa (Additional File 1: Supplementary Fig. S2B), consistent with the predicted molecular weight of the fusion protein. Western blot analysis using anti-GST monoclonal antibodies further validated the identity of the purified recombinant protein (Additional File 1: Supplementary Fig. S2C).

**Fig. 5:**
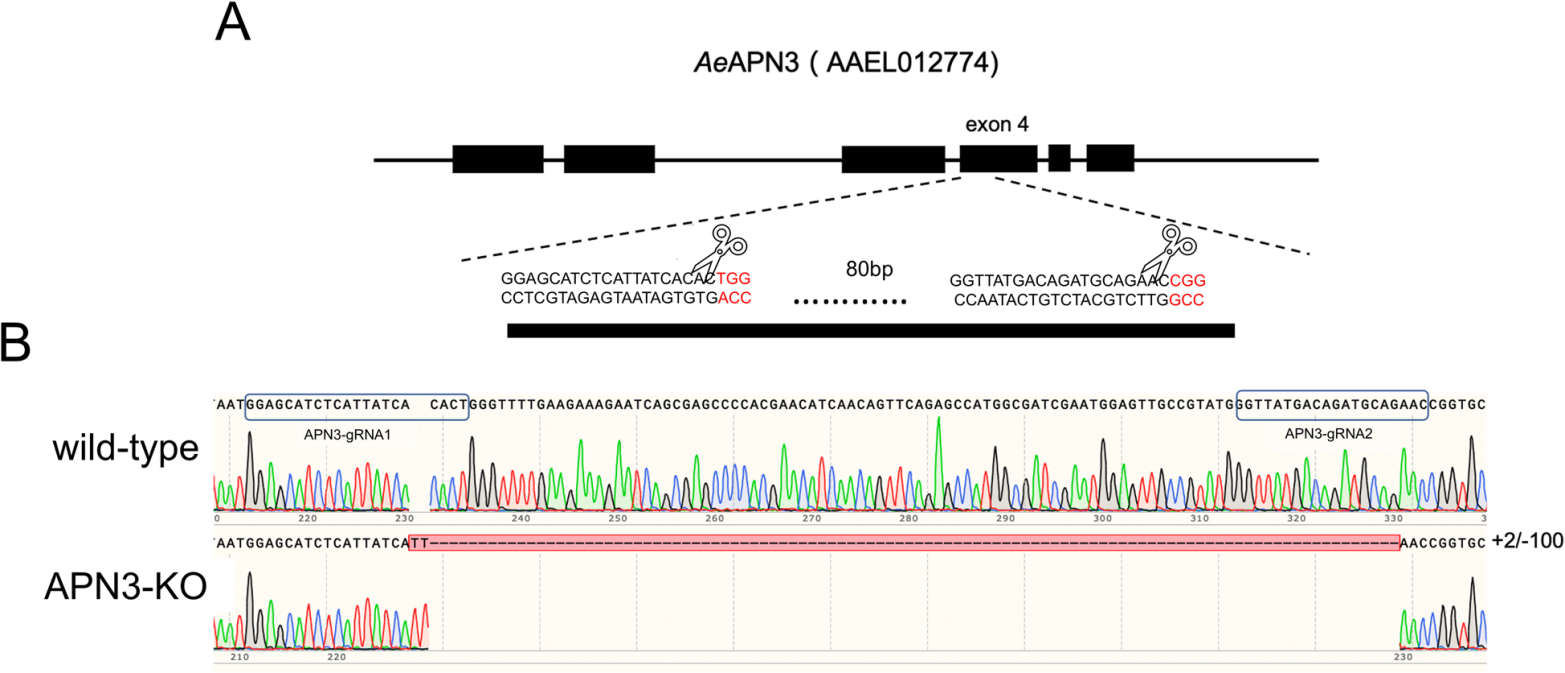
In vitro binding affinity of GST–APN3 protein to Cry4Ba protein. **A** Ligand blot analysis for the interaction between APN3 and Cry4Ba. **B** ELISA analysis for the binding affinity of APN3 to Cry4Ba

The interaction between AeAPN3 and Cry4Ba protein was qualitatively analyzed through complementary ligand-binding methodologies. Ligand blot analysis validated specific binding bands corresponding to the APN3–GST fusion protein and Cry4Ba protein, providing preliminary evidence for direct protein–protein interaction between the purified recombinant AeAPN3 and the Cry4Ba protein (Fig. 5a). Furthermore, quantitative validation through the ELISA binding assay demonstrated a dissociation constant (*K*_*d*_) of 20.53 nM, which suggested a high affinity between the Cry4Ba protein and recombinant AeAPN3 protein (Fig. 4b). These results suggested that AeAPN3 is a putative binding receptor of Cry4Ba.

### Establishment of a homozygous AeAPN3 knockout strain

To knock out AeAPN3, we injected a mixture of two single guide RNAs (sgRNAs) targeting exon 4 of *AeAPN3* and Cas9 protein into freshly laid eggs from an exuCas9-kmo knock-in strain. This knockout site was chosen because it was upstream of the GPI anchor, thus preventing protein function with any frameshift mutation. A total of 200 freshly laid eggs were consecutively injected, of which approximately 6% (12/200) successfully hatched, and among them, 66.7% (8/12) larvae eventually developed into adults (Additional File 1: Supplementary Table S3). Successful site-specific mutations within *AeAPN3* were identified in all surviving G0 mosquitoes (8/8) by sequencing PCR products from individual DNA samples (Additional File 1: Supplementary Table S3).

**Fig. 6:**
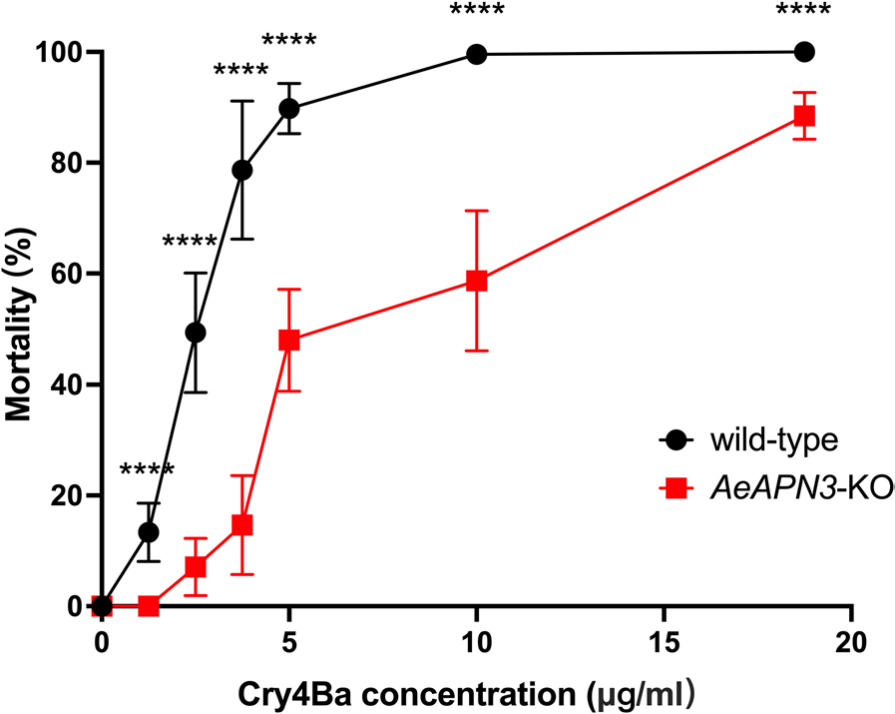
Generation of* AeAPN3* knockout *Ae. Aegypti* strains. **A** Schematic representations of the* AeAPN3* locus, with the third exon containing 20-nucleotide sgRNA target sequences, and the PAM sequence shown in red. **B** Aligned Sanger-sequencing trace of PCR-amplified genomic DNA from wild-type and *AeAPN3*-KO strains with specific primers (Additional File 1: Supplementary Table S1) spanning the genomic RNA-targeted region

The homozygous knockout (KO) *Ae. aegypti* strain for *AeAPN3* (named *AeAPN3*-KO-EXU) was generated using a reverse genetics approach. *AeAPN3*-KO-EXU presented a 100-base pair (bp) deletion and 2 bp insertion between two of the CRISPR/Cas9 target sites (Fig. 6), which should result in truncated protein production (Additional File 1: Supplementary Fig. S3). To eliminate the influence of endogenous expression of the CRISPR/Cas9 system and *kmo* gene deletion on subsequent functional verification, red-fluorescent and white-eyed individuals were removed from *AeAPN3*-KO-EXU strains by fluorescent screening and gene sequencing. Consequently, a new homozygous knockout strain for *AeAPN3* named *AeAPN3*-KO was established by selecting individuals with nonfluorescent normal eyes. No visual phenotype was observed.

### Resistance to Cry4Ba protoxin caused by AeAPN3 knockout

Susceptibility to the Cry4Ba and Cry11Aa protoxins using six gradient concentrations was tested in the *AeAPN3*-KO strain with the original susceptible wild-type strain as a negative control. Bioassay results showed a lethal concentration 50% (LC_50_) value of 7.275 [95% confidence interval (CI): 6.672–7.986] μg/mL to the Cry4Ba protoxin for *AeAPN3*-KO, which was approximately threefold higher than the susceptible wild-type strain [2.476 (95% CI: 1.753–3.175) μg/mL] (Table 1). This result indicated that AeAPN3 is a functional receptor for Cry4Ba. However, for bioassays of Cry11Aa protoxin, the LC_50_ value of the *AeAPN3*-KO strain [0.521 (95% CI: 0.473–0.568) μg/mL] was lower than the wild-type strain [0.801 (95% CI: 0.729–0.876) μg/mL] (Table 1), which indicated that the *AeAPN3*-KO strain became more sensitive to Cry11Aa.

**Table 1:** Susceptibility of *Ae. aegypti* strains to Cry4Ba and Cry11Aa protoxins

Protoxin	Strains	*n*	Slope (SE)	LC_50_ (μg/mL) (95% CI)	LC_50_ ratio
Cry4Ba	Wild-type	1350	3.023 (0.159)	2.476 (1.753–3.175)	1
	*AeAPN3*-KO	1350	2.953 (0.142)	7.275 (6.672–7.986)	2.938
Cry11Aa	Wild-type	1350	2.251 (0.12)	0.801 (0.729–0.876)	1
	*AeAPN3*-KO	1350	2.291 (0.122)	0.521 (0.473–0.568)	0.650

**Fig. 7:**
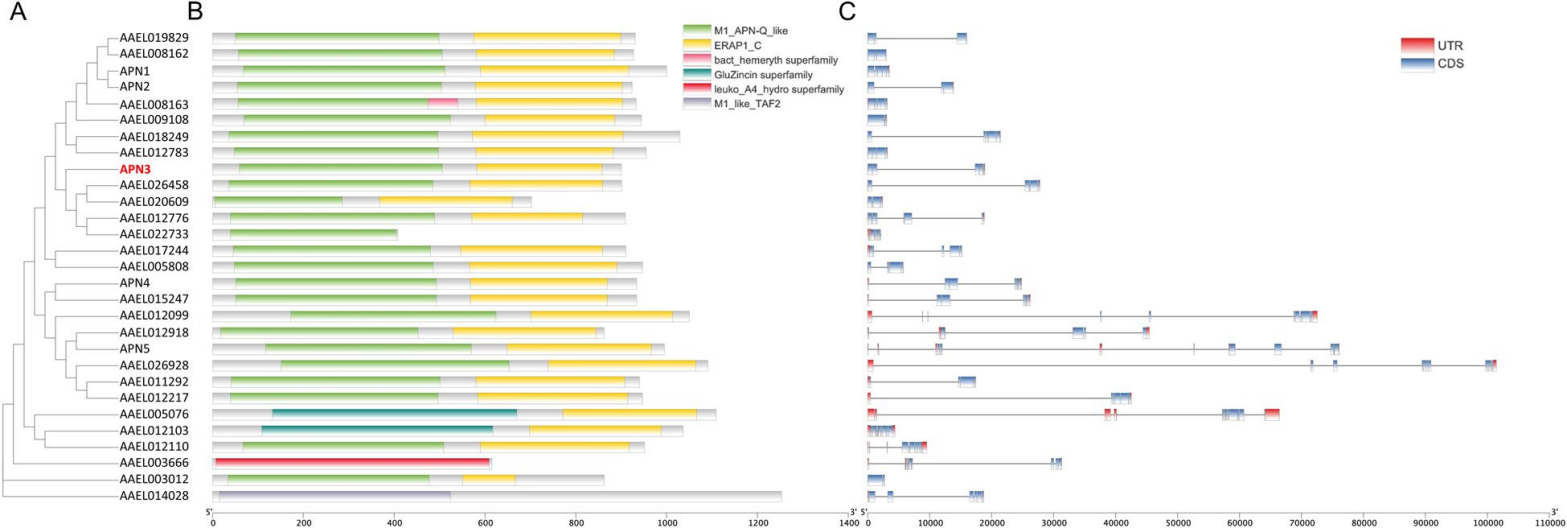
Response of the *AeAPN3*-KO and wild-type strains to Cry4Ba protoxin. A total of 25 larvae from wild-type and *AeAPN3*-KO strains were placed in cups containing six different concentrations of Cry4Ba protoxin. Larval mortality was assessed after 24 h. Each experimental setup included three replicates, and three independent experiments were performed in total. Each point represents the mean ± standard deviation (SD) from three biological replicates. Differences between groups were analyzed by the Mann–Whitney *U* test (*U*_(18)_ = 0, *Z* = 0, ^****^P < 0.0001)

To elucidate the role of AeAPN3 in response to Cry4Ba, we conducted a comparative analysis of mortality rates between the *AeAPN3*-KO and wild-type strains. Our results demonstrate that the mortality rate of *AeAPN3*-KO was significantly lower than that of wild-type across all tested Cry4Ba concentrations (*U*_(18)_ = 0, *Z* = 0, *P* < 0.0001, Mann–Whitney *U* test). Specifically, at the LC_50_ concentration for wild-type (2.5 μg/mL), the mortality rate for *AeAPN3*-KO was only 7.1 ± 5.2%. Furthermore, at a diagnostic dose (10 μg/mL) of Cry4Ba for wild-type strains, the mortality rate for *AeAPN3*-KO increased to 58.7 ± 12.7% (Fig. 7). Notably, when Cry4Ba concentration was further elevated to 18.75 μg/mL, there was a corresponding increase in mortality rate observed in *AeAPN3*-KO mosquitoes, reaching 88.4 ± 4.3%. These findings indicated that AeAPN3 plays a significant role in mediating the activity of Cry4Ba at low lethal concentrations (Fig. 7).

## Discussion

As a class of proteolytic enzymes, APN (EC3.4.11) is primarily distributed in the intestinal and salivary glands of insects, playing a crucial role in protein and peptide digestion by effectively cleaving N-terminal amino acid residues [31]. Eight to ten *APN* genes have been found in the genomes of Lepidopteran insects such as *Bombyx mori*, *Helicoverpa armigera*, *Plutella xylostella*, and *Spodoptera frugiperda *[31]. Additionally, 5 *APN* transcripts were identified in the transcriptome in the *Nilaparvata lugens* of Hemiptera [32], while 11 APN transcripts were discovered in the midgut transcriptome from *Chrysomela populi* of Coleoptera [33]. Notably, 29, 25, and 25 *APN* genes were exhibited in the genomes of three major pathogenic mosquitoes *Ae. aegypti*, *Cu. quinquefasciatus*, and *An. Gambiae*, respectively (Fig. 2). The present finding suggests a pronounced expansion trend of the *APN* gene family in Diptera mosquitoes compared with other insect orders (Figs. 2 and 7). However, by analyzing the spatiotemporal transcriptome of *Ae. aegypti*, our investigation identified ten APN transcripts that remained constitutively silent or exhibited minimal expression throughout ontogenetic development, suggesting that the *APN* family in *Ae. aegypti* may have undergone duplication events resulting in nonfunctional pseudogenes (Fig. 4). Through physical mapping, we observed that while *Ae. aegypti* *APN*s were distributed across three chromosomes, they predominantly clustered in the upper and lower regions of chromosome 1 (Fig. 1). This clustering pattern was also evident in Lepidopteran *APN*s, such as *APN1/APN3*, APN2/APN4/APN6, and *APN7/APN9*, which are arranged adjacent to each other on the genome of *He. armigera*, indicating that gene duplication events of *APN*s mainly occur in close proximity [34]. Through the investigation of genetic evolution in five Lepidoptera species, it was discovered that there exists collinearity among *APN* genes across these different orders. The arrangement and orientation of *APN* gene groups or species within the genome exhibit a general consistency, indicating a conservation of the *APN* gene family during evolutionary processes in Lepidoptera [34]. However, the genetic evolutionary patterns of mosquito *APN*s show significant differences in *Ae. aegypti*. While *AeAPN1*, *AeAPN3*, and AAEL012776 are closely located on the chromosome, they exhibited substantial variations in gene structure and belong to two different major branches, reflecting the diversity in expansion evolution of the mosquito *APN* gene family (Figs. 1, 2 and 3).

The APN protein is predominantly expressed in both a membrane-bound and soluble form. In the insect gut, the membrane-bound APN protein attaches to the brush border membrane of epithelial cells through its GPI anchor site at the C-terminus. In mammals, GPI-APNs have been identified as membrane receptors for various pathogens [35–38]. Conversely, in insects, GPI APNs were primarily acknowledged as crucial receptors for Bt Cry toxins and played significant roles in both toxicological responses and resistance mechanisms against Bt toxins [18, 22]. Furthermore, it was discovered that APN functions as a receptor protein for pea enation mosaic virus (PEMV) and lectin proteins in pea aphids (*Acyrthosiphon pisum*) [39, 40]. In this study, 11 APNs of *Ae. aegypti* were predicted to have GPI anchor sites, including Bti-binding proteins APN1, APN2, APN4, and APN5 (Supplementary Table S2), using three different GPI prediction software tools (big-PI Predictor, PredGPI predictor, and KohGPI). However, Chen et al. also identified a potential GPI anchor site on APN3 using DGPI software, which suggests variations in prediction results among different algorithms [15]. Therefore, the knockout site we chose is located at their predicted GPI anchor site.

Some studies revealed the significance of glycosylation sites in APN for its binding process with Cry proteins [41]. In Bti-binding proteins APN1–5 of *Ae. aegypti*, a number of O-glycosylation and N-glycosylation sites were predicted (Additional File 1: Supplementary Table S2). However, it should be noted that glycosylation is not necessarily the key mediator of the interaction between APN and Cry toxins. Several insect APNs expressed in prokaryotic systems also exhibited specific binding abilities to Cry toxins. For instance, fragments Ile135-Pro198 and Ile135-Gly174 from BmAPN1 expressed in a prokaryotic system retained full binding ability to Cry1Aa, while fragment Gly155-Pro198 retained partial binding capacity [42]. Although AeAPN3 was found to specifically bind to Cry11Aa through GST-pull down and Co-IP experiments, it was not identified as a Cry4Ba-binding protein through mass spectrometry analysis [14]. To further investigate the interaction between AeAPN3 and Cry4Ba, we cloned the full-length *AeAPN3* gene into the PET32a vector for prokaryotic expression. Following IPTG induction, His-tagged AeAPN3 protein predominantly formed inclusion bodies within precipitates. To enhance the solubility of the AeAPN3 protein, we introduced AeAPN3 into the pCold-GST vector for expression. Results revealed that GST-tagged APN3 was present both in supernatant and precipitate fractions. The soluble form of AeAPN3 with a GST tag was purified using a GST chromatography column (Additional File 1: Supplementary Fig. S2). Ligand blotting and ELISA further confirmed a strong interaction between APN3 and Cry4Ba with an affinity of 20.53 nM. This indicated that APN3 is a membrane-bound protein for both Cry4Ba and Cry11Aa, while suggesting that their interaction may not rely on glycosylation modifications (Fig. 5). Some studies observed similar results in interactions between AeAPNs, such as AeAPN1 [15] or AeAPN2a [16], with Bt toxins.

Although APNs have been confirmed as the major binding proteins for Bt toxins in numerous insect species, it should be noted that not all APNs are capable of binding to Cry proteins. Even if APN proteins could bind with Cry proteins, it does not necessarily mean that they are functional receptors mediating Cry activity. In *He. armigera*, ligand blot analysis revealed that HaAPN1 could bind to Cr1A, and overexpression of HaAPN1 in Sf21 insect cells caused aberrant cell morphology [17]. However, CRISPR/Cas9-mediated knockout of *HaAPN1*, *HaAPN2*, and *HaAPN5* did not affect the sensitivity of larvae to Cry1A and Cry2A, likely excluding a major contribution of these genes to Bt toxicity mechanisms [23]. Similarly, GST-pull down and Co-IP experiments showed that* AeAPN1* could specifically bind to Cry4Ba and Cry11Aa, while AeAPN2 could specifically bind to Cry11Aa in *Ae. Aegypti *[14]. Nevertheless, subsequent bioassays conducted on both wild-type strains and CRISPR/Cas9-generated knockout strains lacking either *AeAPN1* or *AeAPN2* showed no change in sensitivity toward these two types of Cry toxins [22]. These findings suggest that neither AeAPN1 nor AeAPN2 serve as essential functional receptors for Bti [22]. This finding contrasts with the results reported by Saengwiman et al., who used RNA interference (RNAi) to knock down *AeAPN1 * [43]. The discrepancy may arise because their double-stranded RNA (dsRNA) targeted conserved domains of ANP isoforms, which share high sequence and structural homology, potentially silencing multiple APN genes simultaneously. In contrast, CRISPR/Cas9 targeting genomic DNA can effectively avoid this issue and the problem of dsRNA degradation in oral RNAi delivery. Thus, we employed CRISPR/Cas9 to knock out ANP3 in this study. However, knocking out *AeAPN3* resulted in approximately threefold higher relative resistance to Cry4Ba, indicating the potential involvement of AeAPN3 in mediating Cry4Ba functionality. In this experiment, *AeAPN3* knockouts seemed to be more sensitive to Cry11Aa, but there is no significant difference compared with the previous wild-type bioassay test [22].

The mechanism of Bt action is highly intricate and may involve the mediation of multiple membrane-bound receptors. In the diamondback moth, knockout of either *PxABCC2* or *PxABCC3* did not result in significant Cry1Ac resistance, whereas simultaneous knockout of both genes led to a remarkable 8000-fold increase in Cry1Ac resistance in larvae [44, 45]. These findings indicate the redundant role of PxABCC2 and PxABCC3 as functional receptor proteins mediating Cry1Ac activity [44, 45]. In *Ae. aegypti*, knock out of *AeCAD *[46] and *AemALP *[47] separately or simultaneous knockout of *AeAPN1* and *AeAPN2 *[22] had no impact on sensitivity to Cry4Ba and Cry11Aa. However, results from *AeAPN3*-KO experiments suggest that *AeAPN3* played a more prominent role in the toxic mechanism of Cry at low concentration. At high concentration, Cry proteins may exert toxicity through multiple receptors or pathways (Fig. 7). In the classical Bt model, activated Cry proteins first form oligomers by interacting with CAD before binding GPI-anchored proteins (APN and ALP) on the cell membrane surface, causing perforation leading to cell death [48]. The role of APN may be similar to that of a “magnet,” absorbing free Cry onto the cell membrane. Subsequently, enriched Cry binds with other functional receptors (such as ABC transporters) to complete the perforation process. Therefore, low doses of Cry require mediation by binding receptors such as APN. However, once the concentration reaches a certain threshold level, direct interaction between Cry and other functional receptors can occur without APN absorption.

## Conclusions

Overall, through ligand blot and ELISA analyses, this study demonstrated that AeAPN3 functions as a high-affinity binding receptor for the Cry4Ba protein. Gene knockout experiments further confirmed that *AeAPN3* depletion significantly enhanced resistance to Cry4Ba protein, thereby establishing AeAPN3 as the functional receptor mediating Cry4Ba toxicity.

## Supplementary information

## Supplementary Information

**Additional file 1.** **Table S1.** PCR primers. **Table S2**. Gene feature profiling of APNs in *Ae. Aegypti.* **Table S3**. Injection, survival, and mutagenesis rates mediated by CRISPR/Cas9 constructs in the ExuCas9-kmo strain. **Fig. S1**. Multiple alignments of specific motifs in *AeAPNs*. **Fig. S2**. Prokaryotic expression of GST-APN3 protein. **Fig. S3.** Amino acid sequence alignment of APN3 isoform from wild-type and APN3-KO strains.

## Data Availability

Data supporting the main conclusions of this study are included in the manuscript and supplementary material.
